# The Potential Impact of Inducing a Restriction in Reimbursement Criteria on Vitamin D Supplementation in Osteoporotic Patients with or without Fractures

**DOI:** 10.3390/nu14091877

**Published:** 2022-04-29

**Authors:** Luca Degli Esposti, Valentina Perrone, Stefania Sella, Gaetano Arcidiacono, Francesco Bertoldo, Andrea Giustina, Salvatore Minisola, Nicola Napoli, Giovanni Passeri, Maurizio Rossini, Sandro Giannini

**Affiliations:** 1 CliCon Srl Società Benefit Health Economics and Outcome Research, 40137 Bologna, Italy; luca.degliesposti@clicon.it (L.D.E.); valentina.perrone@clicon.it (V.P.); 2 Clinica Medica 1, Department of Medicine, University of Padova, 35128 Padova, Italy; stefania.sella@unipd.it (S.S.); gparcidiacono91@gmail.com (G.A.); 3 Internal Medicine, Department of Medicine, University Hospital AOUI, 37134 Verona, Italy; francesco.bertoldo@univr.it; 4 Institute of Endocrine and Metabolic Sciences, Instituto di Ricovero e Cura a Carattere Scientifico (IRCSS) San Raffaele Hospital, San Raffaele Vita-Salute University, 20132 Milan, Italy; giustina.andrea@hsr.it; 5 Department of Clinical, Internal, Anaesthesiology, and Cardiovascular Sciences Sapienza University of Rome, 00185 Rome, Italy; salvatore.minisola@uniroma1.it; 6 Division of Endocrinology and Diabetes, Università Campus Bio-Medico di Roma, 00128 Rome, Italy; n.napoli@unicampus.it; 7 Unit of Clinica e Terapia Medica, Department of Medicine and Surgery, University of Parma, 43126 Parma, Italy; giovanni.passeri@unipr.it; 8 Rheumatology Unit, Department of Medicine, University of Verona, 37134 Verona, Italy; maurizio.rossini@univr.it

**Keywords:** osteoporosis, vitamin D supplementation, regulatory restriction, refracture risk, clinical setting

## Abstract

In October 2019, the Italian Drug Agency (AIFA) restricted reimbursement criteria for vitamin D (VD) use outside the osteoporosis setting (Note 96). However, whether this restriction could also have involved patients at risk for or with osteoporotic fractures has not yet been investigated. We retrospectively analyzed databases from five Italian Local Health Units. Patients aged ≥50 years with either at least one prescription for osteoporosis treatment or with fragility fractures and evidence of osteoporosis from 2011 to 2020 were included. The proportion of subjects with an interruption in VD treatment before and after the introduction of the new reimbursement criteria and predictors of this interruption were analyzed. A total of 94,505 patients (aged 69.4 years) were included. Following the introduction of Note 96, a 2-fold (OR 1.98, 95% CI: 1.92–2.04) increased risk of VD discontinuation was observed. These findings were independent of seasonal variation, osteoporosis treatment patterns, as well as other confounding variables. However, a higher rate of interruption was observed in patients without vertebral/femur fracture (37.8%) vs. those with fracture (32.9%). Rheumatoid arthritis, dyslipidemia and previous fracture were associated with a lower risk of VD interruption, while stroke increased the risk of VD interruption. Our results highlight that a possible misinterpretation of newly introduced criteria for reimbursement restrictions in VD outside of osteoporosis have resulted in an inadequate level of VD supplementation in patients with osteoporosis. This undertreatment could reduce the effect of osteoporosis therapies leading to increased risk of negative outcome.

## 1. Introduction

Vitamin D (VD) plays a critical role in the homeostatic regulation of calcium [1] and reduced intake or low levels of VD can impact upon bone metabolism, leading to increased parathyroid hormone (PTH) secretion and increased bone resorption [2].

VD deficiency is a common condition worldwide, particularly in elderly and osteoporotic individuals [3,4] and the link between VD deficiency and increased risk of fracture in elderly individuals has been extensively documented in trials, observational studies and meta-analyses [5,6,7,8].

Furthermore, it is also recognized that low levels of serum VD may reduce the positive effect on the bone of several anti-resorptive drugs, with their beneficial action being restored by normalizing VD metabolism [9,10].

In the ICARO study, sub-optimal response to anti-osteoporotic treatment was due to a lack of compliance as well as the absence of calcium and VD supplementation, which increased the risk of fracture in those patients [11]. Evidence from other studies in patients receiving anti-osteoporotic therapy, documents the association between calcium and VD supplementation with reduced mortality following fracture [12,13].

National and international guidelines recommend calcium and VD as add-on supplementation to osteoporosis therapies [14,15]. In clinical practice, the combination of calcium and VD is usually indicated and used for the treatment of osteoporosis in association with drugs characterized by specific anti-fracture activity [16,17].

The Italian Medicines Agency (AIFA) Notes are regulatory documents that define the therapeutic indications for which a certain drug can be reimbursed by the Italian National Health Service (INHS) [18].

In 2017, AIFA correctly recommended the already reimbursed VD supplementation (which at that time was already reimbursable through the national healthcare system) for patients at risk of or with prevalent fragility fracture initiating osteoporotic drugs as outlined in Note 79 [19]. In recent years, an increasing trend in VD prescription and consumption was observed in the Italian general population leading to the possible risk of inappropriate use of VD for the management of clinical conditions outside skeletal fragility-associated disease. To address this, in October 2019 AIFA (via the regulatory recommendation “Note 96”) limited reimbursement for the use of VD (cholecalciferol and calcifediol VD treatment) for the indication of “prevention and treatment of VD deficiency in adult subjects (>18 years of age)” in patients with osteoporosis or bone fragility [20].

In the months following the introduction of Note 96, AIFA reported a 20–40% decrease in reimbursable VD in Italy [21]. However, to date, data are needed to clarify the potential impact of the reduction of VD use, due to the restriction of VD reimbursement, even in osteoporotic patients with and without fragility fracture. In this retrospective real-world study, we evaluated if reduced VD consumption (mainly aimed at restricting VD use outside the osteoporosis setting) could have an impact on VD utilization in patients at risk or with fragility fractures.

## 2. Materials and Methods

### 2.1. Study Design and Data Sources

This retrospective analysis was performed with the specific aim of evaluating whether an amendment to drug reimbursement criteria for VD (Note 96) issued from the national drug agency in Italy (AIFA) may impact VD utilization even in patients at risk of or with fragility fractures [20]. Briefly, these newly introduced reimbursement criteria for VD prescriptions apply to:

(a) Institutionalized people, (b) pregnant or lactating women, (c) osteoporotic/osteopenic patients not eligible for remineralizing therapy, regardless of the determination of 25-hydroxyvitamin D levels (25(OH)D) and, upon measurement of 25(OH)D, (d) patients with a 25(OH)D level <20 ng/mL and symptoms related to hypovitaminosis, (e) patients with hyperparathyroidism secondary to hypovitaminosis D, (f) osteoporotic patients eligible for remineralizing therapy that could benefit from hypovitaminosis correction prior to the start of therapy and (g) patients treated with drugs that interfere with the metabolism of VD or patients suffering from conditions causing malabsorption.

A full description of Note 96, as issued by AIFA, is available as Supplementary Material (Supplementary Material Box S1).

Administrative databases of five local health units (LHUs) geographically distributed across Italy covering approximately 2.6 million subjects (approximately 4% of the Italian national population) were retrospectively analyzed. These administrative databases are large repositories of data on healthcare systems that are routinely collected and include INHS provided healthcare services. Therefore, they hold information intended to be used for administrative purposes in order to track the economic flow from the INHS to the healthcare provider for reimbursement purposes. Main dataflows concern: demographic registries, to collect information on age, gender and death; pharmaceutical database, with direct and indirect distribution flow providing data on prescription as anatomical-therapeutic chemical (ATC) code, number of packages, number of units per package, unit cost per package and prescription date; hospital discharge records, that includes all hospitalization data with discharge diagnosis codes, classified according to the International Classification of Diseases, Ninth Revision, Clinical Modification (ICD-9-CM), diagnosis Related Group (DRG) and DRG-related charge (provided by Health System); outpatient specialist care services database, which contains date of prescription, type, description activity of diagnostic tests and visits for patients in analysis and laboratory test or specialist visit charge. To guarantee patient privacy, an anonymous univocal numerical code was assigned to each subject included in the study, in full compliance with the European General Data Protection Regulation (2016/679). All results derived from this analysis were produced as aggregated summaries, which could not be connected, either directly or indirectly, to individual patients. Informed consent was not required since obtaining it is impossible for organizational reasons (pronouncement of the Data Privacy Guarantor Authority, General Authorization for personal data treatment for scientific research purposes–n.9/2014). This study has been notified and approved by the local Ethics Committee of the LHUs involved in the study.

### 2.2. Patients

**Figure 1:**
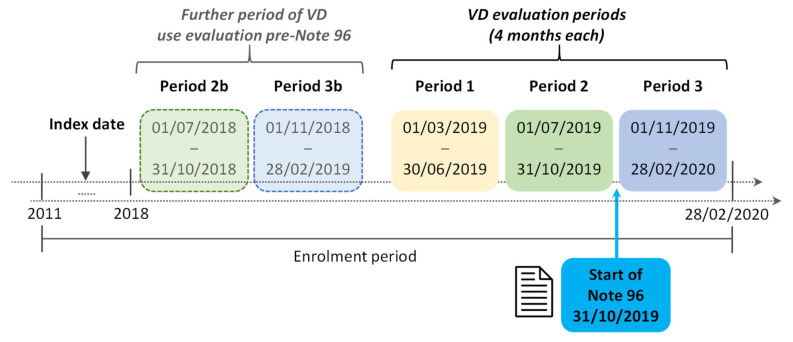
Study periods for the evaluation of VD use and interruption. VD = vitamin D.

All patients aged ≥50 years were included in this analysis if they met one of the following inclusion criteria from 1 January 2011 to 28 February 2020 (enrolment period): (i) at least one prescription for any drugs reimbursed in Italy for the treatment of osteoporosis [bisphosphonates, ATC code: M05BA (ATC code: M05BA08- 1F 4 mg and 100 mg excluded), bisphosphonate combinations (ATC code: M05BB), other drugs affecting bone structure and mineralization (ATC code: M05BX, of which denosumab, ATC M05BX4), parathyroid hormones and analogues (ATC code: H05AA), calcitonin preparations (ATC code: H05BA), selective estrogen receptor modulators (ATC code: G03XC)]; (ii) at least a primary discharge diagnosis of either (a) fracture of the vertebral column without mention of spinal cord injury (ICD-9-CM code: 805); (b) fracture of vertebral column with spinal cord injury (ICD-9 806); (c) fracture of the femoral neck (ICD-9-CM code: 820) with a replacement procedure code (ICD-9-CM code: 79.00, 79.05, 79.10, 79.15, 79.20, 79.25, 79.30, 79.35, 79.40, 79.45, 79.50, 79.55; 81.51, 81.52) with evidence of osteoporosis at baseline (prescription for osteoporosis drugs or hospitalization for osteoporosis with ICD-9-CM code 733.0). The first match with an inclusion criterion during enrolment period was considered as the index date (Figure 1). Patients remained included in the analysis regardless of the presence of osteoporosis treatment during follow-up. Patients were excluded if they had records for renal disease (ICD-9-CM: 584–585 or exemption code 023) and/or cancer (ICD-9-CM code 140–208 or exemption code 048) in the year prior to inclusion or if they died during the follow-up period, which was from the index date to 28 February 2020. Comorbidity profile and co-treatments of patients at baseline were evaluated one year before the index date (characterization period) as the presence of specific ICD-9-CM codes or ATC codes as reported in Supplementary Table S1.

The VD supplementations evaluated were: VD and analogues (all the molecules comprised in the ATC code A11CC except for alfacalcidol [ATC code: A11CC03] and calcitriol [ATC code: A11CC04]); calcium, combinations with VD and/or other drugs (ATC code: A12AX).

### 2.3. Definition of Cohorts Analyzed

As depicted in Figure 1 (see right half of Figure 1), among patients included, the presence of VD was evaluated during three distinct four-month periods: (a) all patients with VD supplementation were identified within Period 1 (1 March–30 June 2019); (b) the same was performed in the following four months (Period 2) (1 July–31 October 2019) and the proportion of patients “not on VD Supplementation” (because of the absence of a new prescription or due to its interruption) was compared to Period 1 (interruption pre-Note 96). Similarly, (c) the same was undertaken in the following four months, during Period 3 (1 November 2019–28 February 2020), i.e., following the introduction of Note 96, when the proportion of patients “not on VD supplementation” (because of the absence of a new prescription or due to its interruption) were compared to Period 2 (interruption post-Note 96). To minimize time-period bias, a further sub-analysis was also performed to compare the “not on VD supplementation” post-Note 96 with the “not on VD supplementation” rate of the same months of Period 3 in the previous year (1 November 2018–28 February 2019; Period 3b) among patients prescribed VD during the same months of Period 2 (1 July–31 October 2018; Period 2b) (left half of Figure 1). Patients included for the presence of osteoporosis treatment at index date were maintained in the analysis, regardless of their utilization during follow-up.

### 2.4. Statistical Analysis

Continuous variables are presented as mean ± standard deviation (SD) and categorical variables are expressed as numbers and percentages. Multivariable logistic regression analysis was performed to investigate potential risk factors for the interruption of VD and presented with relative odds ratio (OR) and 95% confidence intervals (95% CI). Variables included in logistic regression models included clinical characteristics and co-treatments reported at baseline. Model calibration was assessed using the Hosmer–Lemeshow test and model discrimination was assessed using the C-statistic (receiver operating characteristic). The Student’s t-test was used to compare continuous variables, and the Chi-square test was used for categorical ones. A *p*-value <0.05 was considered statistically significant and all analyses were performed using STATA SE version 12.0 (StataCorp, Lakeway Drive, College Station, TX, USA).

## 3. Results

### 3.1. Patient Characteristics

A total of 94,505 patients met the inclusion criteria and their characteristics are summarized in Table 1. Mean age was 69.4 years and more than 90% were women. The most frequently reported comorbidities were hypertension (60.2%) followed by dyslipidemia (28.6%), pulmonary diseases and diabetes (approximately 13%). Among co-treatments commonly observed, 44.8% received protein pump inhibitors, 24.3% platelet aggregation inhibitors and 14.6% corticosteroids for systemic use. A total of 47,866 patients during Period 1 and 45,736 during Period 2 were identified with evidence of VD prescriptions. The cohort of Period 2 mainly included all patients from Period 1 that were still on treatment plus incident patients. Therefore, as reported in Table 1, the two cohorts shared almost the same clinical characteristics and comorbidity profile as well as the same concomitant treatment pattern.

### 3.2. Vitamin D Interruption Rate before and after the Application of Note 96

**Figure 2:**
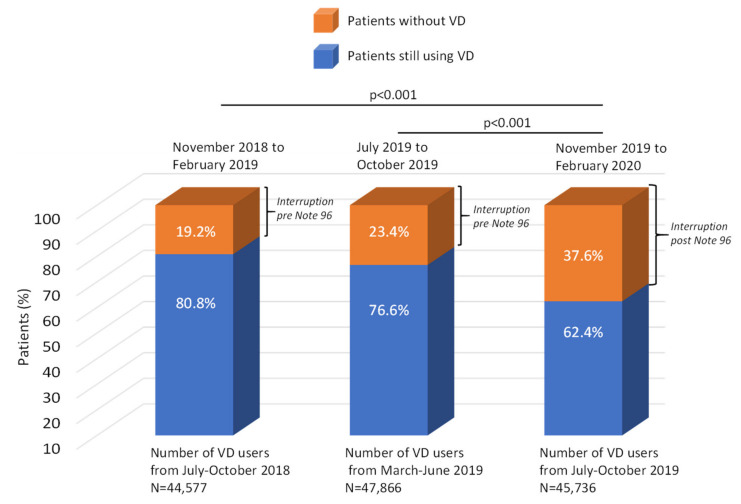
Rate of interruption of VD supplements before and after the application of Note 96. VD = vitamin D.

Among patients with evidence of VD prescriptions during Period 1, the proportion of interruption (or not initiating VD treatment) pre-Note 96 was 23.4% (Figure 2), and similar interruption rates were reported in both patients with and without vertebral or femur fractures before Period 2 when these two sub-groups were considered separately (Table 2). The proportion of patients with an interruption was higher among patients aged >90 years (30%), while lower values (22–25%) were observed in the other age groups. Considering the total population (*n* = 94,505), compared to Period 1 (*n* = 47,866), an increased interruption rate was observed post-Note 96, with 37.6% of VD users of Period 2 (*n* = 45,736) discontinuing/not on VD supplementation during Period 3 (*p* < 0.001) (Figure 2). This was slightly more evident among patients without vertebral or femur fractures (37.8%) than in those with fractures (32.9%) and among patients aged >90 years (47.2%) (Table 2). Characteristics of patients in Period 3 are reported in Supplementary Table S2.

### 3.3. Predictors of Vitamin D Interruption

To further explore potential risk factors associated with VD interruption, logistic regression analysis was performed. The presence of comorbidities such as rheumatoid arthritis (OR 0.82, 95% CI: 0.73–0.92), dyslipidemia (OR 0.92, 95% CI: 0.89–0.95) and previous fracture (OR 0.91, 95% CI: 0.83–0.99) were associated with a lower risk of VD interruption, while stroke (OR 1.21, 95% CI: 1.02–1.42) represented a positive predictive factor (Table 3). After the introduction of Note 96, compared to the cohort analyzed in Period 1, patients in Period 2 were observed to have a two-fold increased risk of discontinuing or not being on VD supplementation (OR 1.98, 95% CI: 1.92–2.04). In order to avoid the potential bias arising from the periods of selection or seasonal variation, a subsequent analysis was performed to compare the interruption rate of VD (i.e., patients not on VD supplementation) post-Note 96 considering the same months of Period 2 and 3 from the previous year. Specifically, 44,577 patients receiving VD were identified from 1 July–31 October 2018 (characteristics and co-treatments are reported in Supplementary Table S3), of which 19.2% did not have any VD prescription in the period between 1 November 2018 and 28 February 2019, thus confirming a trend of high rate of interruption or not initiating VD treatment after the application of Note-96 (Figure 2).

Additional analysis was performed to evaluate whether a reduction in the use of osteoporosis drug treatment could act as a possible driver for changes in VD use during the same period. Results from this analysis revealed that osteoporosis treatment actually increased slightly over this period (77.3% in Period 1 vs. 79.6% in Period 2) (Table 4), thereby not justifying the observed decrease in VD treatment after Note 96. The characteristics of patients receiving osteoporosis treatments are summarized in Supplementary Table S4.

## 4. Discussion

VD exerts a crucial role in bone mineralization and its deficiency can lead to an increased risk of fragility fractures, frequently observed in patients with osteoporosis [22]. VD supplementation has been shown to prevent systemic bone loss following fracture and decreases the risk of fragility fracture [22].

In Italy, AIFA notes define the reimbursement criteria for all pharmaceuticals products and are periodically updated on the basis of new scientific evidence and the needs emerging derived from daily medical practice. Note 79, established several years ago and still valid, even regulates the pharmaceutical management of osteoporosis in terms of primary or secondary prevention, providing the reimbursement criteria of anti-osteoporosis treatments, including VD supplementations as a specific need for patients treated for the prevention of fracture risk. The marked increase in VD prescriptions documented in previous years [23,24] prompted the introduction of Note 96. This Note defines the reimbursable criteria for access to VD to the specific indication for osteoporosis *per se*, with the aim of maintaining the economic equilibrium of INHS without exceeding expenditure outer limits. Accordingly, after Note 96 was issued in October 2019, a reduction in VD consumption [25], especially in women 40–60 years old (but also involving older male and female subjects) was observed [21].

In the present study, we evaluated whether the restriction of reimbursement criteria for VD prescriptions could have affected even the osteoporotic population in which VD use is warranted from Note 79, by using data from the real-world setting. We have reported a decrease in the rate of VD supplementation among osteoporotic patients, which were around 14–18% higher compared to rates prior to the release of Note 96. Furthermore, the risk of interrupting or not initiating VD was increased two-fold for patients analyzed during the period after the introduction of Note 96. Considering different time periods also revealed that our results were not affected by seasonal variation. Moreover, the reduced trend in VD prescriptions appeared to be independent from the decreased rate of osteoporosis treatment. Indeed, the use of osteoporosis treatments did not show substantial alteration before and after Note 96, suggesting their utilization was not a driving force towards the pattern of VD prescription. Overall, the results of this study highlight a worsening and inadequate level of VD supplementation during osteoporosis treatment, that could reduce the effect of these therapies and lead to increased risk of negative outcome [11,26].

An interesting observation that emerged from our analysis was that the total number of patients on active treatment for drugs on osteoporosis were actually lower (~30,000) compared to the total population that was included in the study (~45,000). This clearly indicates that there is an undertreatment in patients included with fragility fractures, corroborating our earlier findings in a separate analysis [27].

The advantage of prescribing VD supplements in association with osteoporotic drugs has been documented in clinical practice [1,28]. In postmenopausal women treated with osteoporosis drugs in combination with VD supplements, a greater increase in bone density and a more pronounced decrease in fracture risk was observed compared to patients taking only drugs for osteoporosis [26]. In this context, a previous retrospective observational study in a cohort of osteoporotic patients with previous fragility fracture [27] reported a lower incidence rate of refracture per 1000-person years among patients with calcium/VD supplementation compared to those receiving osteoporosis drug only or untreated, as well as a low probability to incur refracture. Furthermore, patients with calcium/VD supplement in addition to osteoporosis drug after a fracture were associated with a 64.4% lower risk of developing a subsequent fracture and with a two-fold lower mortality risk compared to the group receiving osteoporosis drugs only. In parallel with findings for clinical outcomes, the presence of calcium/VD supplementation was also found to be associated with lower mean annual healthcare costs, with hospitalization expenditure accounting for 73.7% and 55.2% of the total cost for the osteoporosis drugs only cohort and osteoporosis drug with calcium/VD cohort, respectively [29]. Overall, evidence from literature highlights the importance of ensuring adequate VD supplementation for the prevention of osteoporotic fragility fracture, to decrease the incidence of these events and to limit the associated economic burden. In this regard, our study suggests the need to optimize VD utilization, particularly for osteoporotic patients and consequently reducing the risk of fracture.

## 5. Study Strengths and Limitations

The strengths of this study are represented by the large sample size of an unselected population performed in the real-life setting that can provide a scenario of the current clinical practice in Italy that yields important insights into the prescribing appropriateness trend in osteoporotic patients.

The limitations of our analysis mainly lie in its descriptive nature, based on data collected through administrative databases. First, since data on the use of VD were retrieved from medical prescriptions, the reasons for VD interruption (or non-initiation) was not retrievable from the databases. Moreover, administrative databases contain data on healthcare resources reimbursed by INHS, therefore out-of-pocket drugs could not be evaluated. Second, the results are representative to the sample population and cannot be generalized to the overall population.

## 6. Conclusions

The present study evaluated whether the modification of reimbursement criteria for VD prescriptions outside osteoporosis could impact on VD utilization, even in osteoporotic patients in Italian real-life clinical practice. Our findings revealed that an increased proportion of osteoporotic patients interrupted (or did not initiate) VD-based therapies (around 38%), and there was a two-fold increased risk of not using VD in the period following the introduction of Note 96. The observed reduction in VD prescription was independent of seasonal variation as well as from osteoporosis treatment patterns. Overall, our study suggests that even if the aim to reduce healthcare costs associated with VD consumption outside the osteoporosis setting is well justified, regulatory recommendations such as Note 96 may lead to potential misinterpretation in real-life clinical practice, as evidenced by a reduction in VD treatment in osteoporotic patients. Careful consideration of the implication and interpretation of these policies is mandatory, in order to optimize the use of VD in these patients and consequently minimize the risk of osteoporotic complications.

## Figures and Tables

**Table 1:** Baseline characteristics of patients.

Characteristics	Overall Patients (*n* = 94,505)	Cohort Period 1 (*n* = 47,866)	Cohort Period 2 (*n* = 45,736)	*p*-Value
Age, years (mean ± SD)	69.4 ± 9.5	68.8 ± 9.5	68.8 ± 9.5	1.000
Female, *n* (%)	86,278 (91.3)	45,557 (95.2)	43,453 (95.0)	0.235
*Comorbidities, n (%)*				
Hypertension	56,878 (60.2)	28,282 (59.1)	27,109 (59.3)	0.561
Diabetes	12,041 (12.7)	5453 (11.4)	5246 (11.5)	0.708
Rheumatoid arthritis	1307 (1.4)	833 (1.7)	803 (1.8)	0.857
Dyslipidemia	27,060 (28.6)	14,143 (29.5)	13,528 (29.6)	0.916
Ischemic heart disease	731 (0.8)	315 (0.7)	301 (0.7)	0.999
Cardiac dysrhythmias	3553 (3.8)	1649 (3.4)	1600 (3.5)	0.656
Heart failure	292 (0.3)	114 (0.2)	104 (0.2)	0.733
Stroke	873 (0.9)	367 (0.8)	326 (0.7)	0.336
Dementia	69 (0.1)	26 (0.1)	27 (0.1)	0.762
Schizophrenic disorders	227 (0.2)	118 (0.2)	104 (0.2)	0.548
COPD	12,127 (12.8)	6195 (12.9)	5970 (13.1)	0.614
*Co-treatments, n (%)*				
Corticosteroids for systemic use	13,792 (14.6)	7365 (15.4)	7073 (15.5)	0.741
Platelet aggregation inhibitors excl. heparin	22,957 (24.3)	11,334 (23.7)	10,888 (23.8)	0.647
VKA/direct factor Xa inhibitors	2774 (2.9)	1259 (2.6)	1207 (2.6)	0.933
Analgesics	7482 (7.9)	3836 (8.0)	3642 (8.0)	0.774
Antiepileptics	5429 (5.7)	2696 (5.6)	2603 (5.7)	0.696
Antipsychotics	1336 (1.4)	572 (1.2)	522 (1.1)	0.445
Proton pump inhibitors	42,367 (44.8)	22,177 (46.3)	21,326 (46.6)	0.362

COPD = chronic obstructive pulmonary disease. VKA = vitamin K antagonist.

**Table 2:** Rate of interruption or non-initiation of VD supplements before and after the application of Note 96 among osteoporotic patients stratified by type of prevention and age distribution.

	VD Treatment Pre-Note 96			VD Treatment Post-Note 96			*p*-Value
Period	Period 1 VD Users	Period 2 VD Users	Period 2 VD Non-Users	Period 2 VD Users	Period 3 VD Users	Period 3 VD Non-Users	
Patient classification	Total number of VD users in March–June 2019	% of patients still using VD in July–October 2019	% of patients not using VD in July–October 2019	Number of VD users in July–October 2019	% of patients still using VD after Note 96 introduction (November 2019–February 2020)	% of patients not using VD after Note 96 introduction (November 2019–February 2020)	
Patients without vertebral or femur fractures	46,454	76.6	23.4	44,334	62.2	37.8	<0.001
Patients with vertebral or femur fractures	1412	76.1	23.9	1402	67.1	32.9	<0.001
*Age distribution*							
50–59	8288	74.3	25.7	7877	60.0	40.0	<0.001
60–69	17,106	77.5	22.5	16,303	63.5	36.5	<0.001
70–79	16,500	77.7	22.3	15,807	63.3	36.7	<0.001
80–89	5643	74.5	25.5	5423	60.3	39.7	<0.001
90+	329	69.6	30.4	326	52.8	47.2	<0.001

Patients analyzed are those with VD prescription during the period considered, regardless of the concomitant presence of osteoporosis treatment. Patients remained included in the analysis independent of the presence of osteoporosis treatment during follow-up. VD = vitamin D.

**Table 3:** Multivariable logistic regression analysis to identify predictors for not using VD in patients evaluated before (Period 1) and after (Period 2) the introduction of Note 96.

Covariates	OR	95% CI		*p*-Value
Age	1.000	0.999	1.002	0.692
Gender (ref. female)	1.355	1.272	1.444	<0.001
Hypertension (ref. absence)	0.989	0.958	1.021	0.488
Diabetes (ref. absence)	1.019	0.973	1.068	0.418
Rheumatoid arthritis (ref. absence)	0.816	0.728	0.915	0.001
Dyslipidemia (ref. absence)	0.916	0.886	0.947	<0.001
Ischemic heart disease (ref. absence)	1.046	0.866	1.264	0.638
Cardiac dysrhythmias (ref. absence)	1.026	0.946	1.113	0.536
Heart failure (ref. absence)	1.004	0.747	1.350	0.978
Stroke (ref. absence)	1.206	1.024	1.420	<0.05
Dementia (ref. absence)	1.257	0.708	2.233	0.435
Schizophrenic disorders (ref. absence)	1.024	0.764	1.371	0.876
COPD (ref. absence)	0.963	0.922	1.006	0.088
Corticosteroids for systemic use (ref. absence)	0.962	0.923	1.004	0.073
Platelet aggregation inhibitors excl. heparin (ref. absence)	0.983	0.946	1.021	0.377
VKA/direct factor Xa inhibitors (ref. absence)	1.142	1.043	1.252	<0.01
Analgesics (ref. absence)	1.000	0.948	1.056	0.990
Antiepileptics (ref. absence)	0.937	0.880	0.999	<0.05
Antipsychotics (ref. absence)	1.168	1.024	1.333	<0.05
Proton pump inhibitors (ref. absence)	0.911	0.883	0.940	<0.001
Previous fractures (ref. absence)	0.905	0.831	0.985	<0.05
Cohort				
Vitamin D treated in P1	1.000			
Vitamin D treated in P2	1.979	1.924	2.036	<0.001

COPD = chronic obstructive pulmonary disease, OR = odds ratio, VKA = vitamin K antagonist.

**Table 4:** Rate of interruption or no initiation of osteoporosis treatments before and after the application of Note 96 among osteoporotic patients overall and stratified by type of prevention.

	Osteoporosis Treatment Pre-Note 96			Osteoporosis Treatment Post-Note 96			*p*-Value
Period	Period 1 Osteoporosis Treatment	Period 2 Osteoporosis Treatment	Period 2 No Osteoporosis Treatment	Period 2 Osteoporosis Treatment	Period 3 Osteoporosis Treatment	Period 3 No osteoporosis Treatment	
Patient classification	Number of osteoporosis treatment users in March–June 2019	% of patients still using osteoporosis treatments in July–October 2019	% of patients without osteoporosis treatments in July–October 2019	Number of osteoporosis treatment users in July–October 2019	% of patients still using osteoporosis treatments after Note 96 introduction (November 2019–February 2020)	% of patients without osteoporosis treatments after Note 96 introduction (November 2019–February 2020)	
Patients with osteoporosis treatment	31,089	77.3	22.7	29,578	79.6	20.4	<0.001
Patients without vertebral or femur fractures	30,241	77.4	22.6	28,768	79.6	20.4	<0.001
Patients with vertebral or femur fractures	848	74.8	25.2	810	79.1	20.9	<0.05

## Data Availability

The data presented in this study are available on request from the corresponding author.

## Supplementary Materials

The following supporting information can be downloaded at: https://www.mdpi.com/article/10.3390/nu14091877/s1, Supplementary Material Box S1: Note 96 as outlined in the Italian Drugs Agency (AIFA) website: https://www.aifa.gov.it/Nota-96 (accessed on 12 April 2022); Supplementary Table S1. Definition of baseline characteristics; Supplementary Table S2: Baseline characteristics of patients in Period 3bis vs. Period 3; Supplementary Table S3: Baseline characteristics of patients in Period 2bis vs. Period 2; Supplementary Table S4: Baseline demographic characteristics, comorbidity profile and co-treatments of VD users during 1 July–31 October 2018.
