# Supplementary material for: The Potential Impact of Inducing a Restriction in Reimbursement Criteria on Vitamin D Supplementation in Osteoporotic Patients with or without Fractures
Source: Nutrients. 2022 Apr 29;14(9):1877. doi: 10.3390/nu14091877 (PMC9105449; doi:10.3390/nu14091877)
Supplement: Supplementary file 1 [file nutrients-14-01877-s001.zip › nutrients-1703015-supplementary.pdf]

Supplementary Material Box 1. Note 96 as outlined in the Italian Drugs Agency (AIFA) website; https://www.aifa.gov.it/Nota-96

The prescription by the NHS of drugs with the indication **"prevention and treatment of vitamin D* deficiency"** in adults (>18 years) is limited to the following conditions:
Prevention and treatment of vitamin D deficiency in the following clinical scenarios:

regardless of measurement of 25(OH)D levels

- institutionalized people
- pregnant or lactating women
- people with osteoporosis of any cause or known osteopathies not candidates for remineralizing therapy

after measuring 25(OH)D levels

- people with serum levels of 25OHD <20 ng/mL and symptoms attributable to hypovitaminosis (asthenia, myalgia, widespread or localized pain, frequent unexplained falls)
- people diagnosed with hyperparathyroidism secondary to hypovitaminosis D
- people with osteoporosis of any cause or known bone disorders candidates for remineralizing therapy for which the

*correction of hypovitaminosis should be a prerequisite for the start of therapy ***

- a long-term therapy with drugs that interfere with vitamin D metabolism
- diseases that can cause malabsorption in adults

Table S1. Definition of baseline characteristics. * Vitamin D drugs included in this note from AIFA include cholecalciferol, cholecalciferol/calcium salt and calcifediol ; ** Remineralizing therapies should be started after correction of hypovitaminosis D.

Characteristics	Definition
Comorbidity	
Hypertension	at least 1 hospitalization with a discharge diagnosis for Hypertensive Disease (ICD-9-CM codes: 401-405) or at least 2 prescriptions of antihypertensive drugs (ATC codes: C03; C07; C08; C09) or presence of exemption for hypertension (code 031; A31);
Diabetes	at least 1 hospitalization with a discharge diagnosis for diabetes (ICD-9-CM code: 250) or at least 3 prescriptions

	of antidiabetic drugs (ATC code: A10) or presence of exemption for diabetes (code 013);
Rheumatoid Arthritis	at least 1 hospitalization with a discharge diagnosis for rheumatoid arthritis and other inflammatory polyarthropathies (ICD-9-CM code: 714) or presence of exemption for rheumatoid arthritis (code: 006);
Dyslipidemia	at least 1 hospitalization with a discharge diagnosis for disorders of lipoid metabolism (ICD-9-CM code: 272) or at least 3 prescriptions of lipid modifying agents (ATC code: C10) or presence of exemption for hypercholesterolemia (code: 025);
Ischemic heart disease	at least 1 hospitalization with a discharge diagnosis for Acute myocardial infarction or Ischemic heart disease (ICD-9-CM codes: 410-414) or presence of exemption for ischemic heart disease (code: A02.414)
Cardiac dysrhythmias	at least 1 hospitalization with a discharge diagnosis for cardiac dysrhythmias (ICD-9-CM code: 427) or at least 2 prescriptions of antiarrhythmics agents (ATC code: C01B) or presence of exemption for cardiac dysrhythmias (code: A02.427)
Heart failure	at least 1 hospitalization with a discharge diagnosis for Heart failure (ICD-9-CM code: 428)
Stroke	at least 1 hospitalization with a discharge diagnosis for Cerebrovascular Disease (ICD-9-CM code: 430-438) or presence of exemption for neurovascular disease (codes: B02.433; B02.434; B02.437)
Dementia	at least 1 hospitalization with a discharge diagnosis for dementias (ICD-9-CM code: 290) or senility without mention of psychosis (icd-9-cm 797) or presence of exemption for dementias (code 011)
Schizophrenic disorders	at least 1 hospitalization with a discharge diagnosis for Schizophrenic disorders (ICD-9-CM codes: 295-299)
Chronic Obstructive Pulmonary Disease and Allied Conditions	at least 1 hospitalization with a discharge diagnosis for Chronic Obstructive Pulmonary Disease and Allied Conditions (ICD-9-CM codes: 490-496) or presence of at least 2 prescriptions of drugs for obstructive airway diseases (ATC code: R03) or presence of exemption for asthma (code: 007.493)
Co-treatments	
Corticosteroids for systemic use	at least 2 prescriptions of corticosteroids for systemic use (ATC code: H02)

Platelet aggregation inhibitors excl. heparin	at least 2 prescriptions of Platelet aggregation inhibitors excl. heparin (ATC code: B01AC)
Vitamin K antagonists/Direct thrombin or factor Xa inhibitors	at least 2 prescriptions of Vitamin K antagonists or Direct factor Xa inhibitors (ATC codes: B01AA; B01AE; B01AF);
Analgesics	at least 2 prescriptions of analgesic agents (ATC code: N02)
Antiepileptics	at least 2 prescriptions of antiepileptic agents (ATC code: N03)
Antipsychotics/anxiolytics	at least 2 prescriptions of antipsychotic or anxiolytics agents (ATC codes: N05A; N05B)
Proton pump inhibitors	at least 2 prescriptions of proton pump inhibitors (ATC code: A02BA).

Supplementary Table S2. Baseline characteristics of patients in Period 3bis vs Period 3.

Characteristics	Cohort Period 3bis (N=46,813)	Cohort Period 3 (N=36,813)	p-value
Age, years (mean±SD)	68.8±9.0	68.7±8.9	0.125
Female, n (%)	44,584 (95.2)	35,016 (95.1)	0.421
Comorbidities, n (%)			
Hypertension	27,475 (58.7)	21,705 (59.0)	0.432
Diabetes	5,337 (11.4)	4,302 (11.7)	0.199
Rheumatoid Arthritis	818 (1.7)	680 (1.8)	0.280
Dyslipidemia	13,824 (29.5)	10,962 (29.8)	0.437
Ischemic heart disease	290 (0.6)	254 (0.7)	0.208
Cardiac dysrhythmias	1,625 (3.5)	1,313 (3.6)	0.457
Heart failure	115 (0.2)	89 (0.2)	0.910
Stroke	362 (0.8)	304 (0.8)	0.396
Dementia	26 (0.1)	20 (0.1)	0.941
Schizophrenic disorders	111 (0.2)	87 (0.2)	0.982
Chronic Obstructive Pulmonary Disease	6,075 (13.0)	4,915 (13.4)	0.112
Co-treatments, n (%)			
Corticosteroids for systemic use	7,274 (15.5)	5,875 (16.0)	0.097

Platelet aggregation inhibitors excl. Heparin	11,135 (23.8)	8,766 (23.8)	0.930
VKA / Direct factor Xa inhibitors	1,219 (2.6)	974 (2.6)	0.707
Analgesics	3,791 (8.1)	3,017 (8.2)	0.610
Antiepileptics	2,730 (5.8)	2,206 (6.0)	0.328
Antipsychotics	559 (1.2)	447 (1.2)	0.791
Proton pump inhibitors	21,709 (46.4)	17,284 (47.0)	0.097

Supplementary Table S3. Baseline characteristics of patients in Period 2bis vs Period 2.

Characteristics	Cohort Period 2bis (N=44,577)	Cohort Period 2 (N=45,736)	p-value
Age, years (mean±SD)	69.0±9.0	68.8±9.5	<0.05
Female, n (%)	42,475 (95.3)	43,453 (95.0)	0.053
Comorbidities, n (%)			
Hypertension	26,515 (59.5)	27,109 (59.3)	0.523
Diabetes	5,099 (11.4)	5,246 (11.5)	0.882
Rheumatoid Arthritis	810 (1.8)	803 (1.8)	0.486
Dyslipidemia	13,263 (29.8)	13,528 (29.6)	0.566
Ischemic heart disease	276 (0.6)	301 (0.7)	0.462
Cardiac dysrhythmias	1,602 (3.6)	1,600 (3.5)	0.438
Heart failure	113 (0.3)	104 (0.2)	0.423
Stroke	355 (0.8)	326 (0.7)	0.147
Dementia	26 (0.1)	27 (0.1)	0.965
Schizophrenic disorders	96 (0.2)	104 (0.2)	0.700
Chronic Obstructive Pulmonary Disease	5,830 (13.1)	5,970 (13.1)	0.910
Co-treatments, n (%)			
Corticosteroids for systemic use	6,983 (15.7)	7,073 (15.5)	0.407
Platelet aggregation inhibitors excl. Heparin	10,771 (24.2)	10,888 (23.8)	0.210

VKA / Direct factor Xa inhibitors	1,192 (2.7)	1,207 (2.6)	0.744
Analgesics	3,668 (8.2)	3,642 (8.0)	0.144
Antiepileptics	2,569 (5.8)	2,603 (5.7)	0.643
Antipsychotics	526 (1.2)	522 (1.1)	0.588
Proton pump inhibitors	20,932 (47.0)	21,326 (46.6)	0.323

Supplementary Table S4. Baseline demographic characteristics, comorbidity profile and co-treatments of VD users during 1 July- 31 October 2018.

Characteristics	VD users during 1 July- 31 October 2018 (N=44,577)
Age, years (mean±sd)	69.0±9.0
Female, n (%)	42,475 (95.3)
Comorbidities, n (%)	
Hypertension	26,515 (59.5)
Diabetes	5,099 (11.4)
Rheumatoid Arthritis	810 (1.8)
Dyslipidemia	13,263 (29.8)
Ischemic heart disease	276 (0.6)
Cardiac dysrhythmias	1,602 (3.6)
Heart failure	113 (0.3)
Stroke	355 (0.8)
Dementia	26 (0.1)
Schizophrenic disorders	96 (0.2)
Chronic Obstructive Pulmonary Disease	5,830 (13.1)
Co-treatments, n (%)	
Corticosteroids for systemic use	6,983 (15.7)
Platelet aggregation inhibitors excl. Heparin	10,771 (24.2)
VKA/direct factor Xa inhibitors	1,192 (2.7)
Analgesics	3,668 (8.2)

Antiepileptics	2,569 (5.8)
Antipsychotics	526 (1.2)
Proton pump inhibitors	20,932 (47.0)
